# The role of primary care in cancer diagnosis via emergency presentation: qualitative synthesis of significant event reports

**DOI:** 10.1038/bjc.2015.42

**Published:** 2015-03-03

**Authors:** E D Mitchell, G Rubin, L Merriman, U Macleod

**Affiliations:** 1 Centre for Health Services Research, Leeds Institute of Health Sciences, University of Leeds, Charles Thackrah Building, 101 Clarendon Road, Leeds LS2 9LJ, UK; 2 Durham University, School of Medicine, Pharmacy and Health, Wolfson Building, Queen's Campus, University Boulevard, Stockton-on-Tees TS17 6BH, UK; 3 North Derbyshire Clinical Commissioning Group, The Springs Health Centre, Recreation Close, Clowne, Chesterfield S43 4PL, UK; 4 Hull York Medical School, University of Hull, Hertford Building, Cottingham Road, Hull HU6 7RX, UK

**Keywords:** emergency presentation, significant event, primary care, qualitative

## Abstract

**Background:**

Patients diagnosed with cancer in the context of an emergency presentation (EP) have poorer outcomes. It is often assumed that such patients present to the emergency department without consulting their general practitioner (GP). Little work has been done to identify primary care involvement before hospital attendance.

**Methods:**

Participating primary care practices completed a significant event audit (SEA) report for the last patient diagnosed with cancer as a result of an EP. Accounts were synthesised and a qualitative approach to analysis undertaken.

**Results:**

SEAs for 222 patients were analysed. A range of cancers were included, the most common being lung (32.4%) and upper gastrointestinal (19.8%). In most cases, patients had contact with their practice before diagnosis, primarily in the period immediately before admission. In only eight cases had there been no input from primary care. Accounts of protracted primary care contact generally demonstrated complexity, often related to comorbidity, patient-mediated factors or reassurance provided by negative investigations. Learning points identified by practices centred on the themes of presentation and diagnosis, consultation and safety-netting, communication and system issues, patient factors and referral guidelines.

**Conclusions:**

There is extensive primary care input into patients whose diagnosis results from EP, and for the most part potential ‘delay' in referral can be reasonably explained by the complexity of the presentation or by coexisting patient factors.

One of the key factors in determining outcomes from many cancers is the route to diagnosis. In the United Kingdom, the main routes to diagnosis are screen detected, urgent ‘2-week wait' (2WW) referral, routine general practitioner (GP) referral, onward referral from another speciality and emergency presentation (EP). In the 2WW pathway (Department of Health, 2000), patients are referred urgently by their GP and can expect to be seen by a specialist within 2 weeks. This is considered to be the gold standard in diagnosis and for many cancers (such as breast, bladder and ovary) results in higher survival compared with presentation by other routes, with the exception of screen-detected cancers (National Cancer Intelligence Network, 2013). Conversely, diagnosis within the context of an EP results in poorer outcomes (McPhail *et al*, 2013). Between 2006 and 2010, 24% of all cancers in England were diagnosed via the emergency route, although there was considerable variation across cancer types (National Cancer Intelligence Network, 2013).

Improving the pathway to diagnosis for patients diagnosed during an EP should improve outcomes, not only for this patient group but also in terms of the United Kingdom as a whole, which is known to have poorer cancer survival than that of comparable countries (Sant *et al*, 2009; Coleman *et al*, 2011). Despite this, we know little about the circumstances and context surrounding the route to EP. Recent work has shown that the majority of patients with a diagnosis of colorectal cancer following EP have seen their GP within the 6 months before diagnosis (Sheringham *et al*, 2014). There is as yet little known about the content of these primary care contacts, and in particular whether there are opportunities for earlier diagnosis within them.

Significant event audit (SEA) is a quality improvement tool designed to assist with and improve the quality of patient care in general practice (Pringle *et al*, 1995; McKay *et al*, 2009). It provides a structured narrative of the events surrounding an incident of interest, and as a tool for self-reflection and improvement is now part of the GP appraisal and revalidation process in the United Kingdom. Yet, although SEA is widely used in primary care practice, its utilisation to obtain insights into the process of care for specific conditions is relatively rare. We have previously used SEA in this novel way and reported an analysis of multiple SEAs of lung cancer diagnosis (Mitchell *et al*, 2013). This paper reports on the analysis of multiple SEAs for patients who were diagnosed with cancer as the result of an EP to hospital. Data were synthesised from four SEA-generating initiatives with the aim of understanding the causes of EP and determining the degree to which earlier intervention by general practice was possible.

## Materials and methods

### Setting

This synthesis used SEAs completed as part of four separate initiatives undertaken by the authors. In 2009, SEAs for lung cancer and cancers in teenagers and young adults (TYA), including EPs, were carried out by practices in the North of England Cancer Network. In 2010, SEAs of upper gastrointestinal (UGI) and ovarian cancers were undertaken by practices in the South East London Cancer Network. Subsequently, SEAs of EP regardless of cancer type were carried out by practices in the North East Yorkshire and Humber Clinical Alliance (Cancer) in 2012 and in the Yorkshire and the Humber Strategic Clinical Network in 2014 (Box 1). These network areas encompass urban, rural and coastal areas, as well as a range of socio-economic backgrounds.

### Data collection

All practices in each area were invited to participate, and those expressing interest were asked to undertake an SEA for the last patient in their practice diagnosed with either the particular cancer under study or as the result of an EP. Practices were provided with a standardised, cancer-specific electronic template for documenting their SEAs. This was developed for and modified following the initial evaluation (Mitchell *et al*, 2009) and was based on the structure recommended by the National Patient Safety Agency (National Patient Safety Agency, 2006). It includes specific prompts designed to give a richer understanding of the circumstances surrounding pathways to diagnosis, and asks practices to consider and reflect on what happened, why it happened, what has been learnt and what can be changed. Completed reports were returned to the local network team, subjected to peer assessment for quality control and anonymised versions forwarded to the research team.

### Data analysis

As each SEA is a narrative account of a new cancer diagnosis and the circumstances surrounding it, qualitative methods, based on a modified framework approach (Ritchie and Spencer, 1994), were used to synthesise findings. Individual SEA documents were read and re-read and a coding frame constructed, which was then applied to the data to identify emerging themes. In addition, an Interpretative Matrix was created to better understand the factors related to the diagnostic pathway. Relevant data from each SEA were extracted and incorporated into a thematic chart to allow identification and interpretation of common and diverse aspects of presentation and pathways of care. QSR Nvivo 2.0 software (Melbourne, VIC Australia) was used to facilitate coding and organisation of data for analysis.

Although analysis of SEAs also has the potential to provide quantitative data related to certain aspects of the diagnostic process, we have focussed on eliciting the context surrounding each presentation and have not quantified data except to characterise participants and identify cases of extended primary care input or longer illness timescales for detailed study.

## Results

### Characteristics of cases

A total of 222 cases of emergency cancer presentation originating from 203 practices were included in the analysis (65.6% of those who participated in the initiatives). Date of diagnosis ranged from 1993 to 2014, with the majority having occurred from 2009 onwards (79.7%). The 1993 diagnosis related to an 18-year-old patient with lymphoma and was returned by the practice as part of an SEA initiative looking at the last TYA diagnosis rather than the last EP specifically. It is therefore possible that this was the most recent case in the practice (Box 1). Average patient age at diagnosis was 65.4 years (s.d. 17.2), and more than half were reported as being alive at SEA completion (Table 1).

The most commonly included cancer site was lung (32.4%), followed by UGI (19.8%) and gynaecological cancers (11.2%). These figures relate to the cancer groups studied during our data collection exercises and are not a reflection of the distribution of cancer diagnoses within a population of emergency presenters. However, the proportion of lung cancer patients who present as emergencies is among the highest for any cancer (National Cancer Intelligence Network, 2013). Nine patients (4.1%) had metastatic disease from a cancer of unknown primary (CUP). Median survival for those patients known to have died and with date of death provided (88.4%) was 39 days (range 0–633 days). The shortest median survival times were found for carcinoid and head and neck cancer (0 and 24 days, respectively), and the longest for brain tumours (136 days). All breast cancer, melanoma and sarcoma patients were alive.

### Symptoms at EP

For many patients, presenting signs were related to the eventual diagnosis, whereas for others symptoms were either not immediately suggestive of cancer or appeared unrelated to the cancer diagnosed (Table 2). Indeed, with the exception of brain tumours and sarcomas (which appeared to manifest only neurological-type signs, and joint pain and swelling, respectively), presenting symptoms involved a range of physiological systems, regardless of tumour type. This is perhaps an indication of the fact that almost half of the included patients were reported as having advanced or metastatic disease at presentation and therefore presented with symptoms related to the metastases rather than to the primary tumour. In addition, a substantial number of patients (50.4%) had multiple symptoms at EP, with only a marginal difference in the numbers related to those reported as having advanced disease at presentation and those without (52.4% and 48.7%, respectively). As was the case with patients whose tumours were identifiable, many of the presenting symptoms for patients with CUP were relatively vague, including abdominal or chest pain and breathlessness.

### Sources of EP

A key finding from analysis of the cases reported is the extent of contact that patients had with primary care, either in relation to the decision to refer as an emergency or in the time immediately before that event (Table 3). In more than half of the 222 cases (52.3%), the emergency admission was arranged by the patient's practice. In an additional 65 cases (29.3%), the practice had been directly involved in managing the episode of illness leading up to the admission, with those patients for the most part subsequently presenting to an emergency department (ED), or being referred there or admitted by other services (including GP out of hours (OOH)). In a further 29 cases (13.1%) the practice had been involved in the care of the patient in the year before diagnosis. In only eight cases (3.6%) could it be established that there had been no input from primary care in the year before diagnosis, and there were an additional two cases (0.9%) for which we were unable to determine whether there had been primary care input.

In 28 of the SEA reports, the information provided was insufficient to determine whether the practice had arranged the emergency admission (or in one case the OOH service) or the patient had self-referred to the ED. As a result, the extent of direct practice involvement in the emergency admission process may be underestimated.

### Understanding primary care input

The time interval between initial presentation with a (related) symptom and emergency admission or ED referral/presentation was identifiable in almost all of the case accounts. Analysis of the SEA reports has established that 51 of the 222 cases (23.0%) had a one-off or short history of contact with the practice, and had the EP arranged by their GP within 2 weeks of the initial consultation. In addition, five patients admitted by OOH, one admitted by the hospital-at-home service, eight patients who presented to the ED and four patients whose source of emergency referral was unclear had short illness timescales in general practice. There were a further eight cases for which we could not discern any primary care contact in the year before EP, and two for which no information on presenting timescale was provided. This leaves 65 patients (29.3%) who had longer contact with the practice before their GP arranged an EP, and 78 cases (35.1%) in which there was input by the practice in the year leading up to the EP, but the source of admission came from elsewhere.

In order to better understand the context surrounding EP, the narratives related to these cases have been reviewed in detail to determine whether there may have been an opportunity for the GP to have intervened before the emergency admission and to have initiated a 2WW referral during the patient's earlier contact with the practice. Four key themes emerged from analysis of these accounts, relating to complexity of presentation, nature of symptoms, patient factors and system issues. In some cases, multiple explanatory factors were at play, while others involved decisions related to patient autonomy or secondary care factors and were consequently beyond the control of the practice team.

### Complexity of presentation

A number of SEAs described complex cases in which it is difficult to see how earlier action might have been taken by the practice. The complexity often related to the differential diagnosis being clouded by coexisting morbidity or to symptoms that initially improved with treatment but became acute before they could be investigated further. There were also many examples of particularly unusual presentations, or of presentations involving relatively straightforward symptoms, which, despite involving a longer period of contact with the practice, demonstrated appropriate patient management by the GP.

### Nature of symptoms

For many of the cases in which the nature of symptoms was particularly relevant, the patient was being investigated by the GP (using diagnostic services, blood or urine tests, or referral to another primary care practitioner such as a physiotherapist), but their condition deteriorated and they were admitted before investigation could be completed and managed referral could be made. In other cases, the GP had safety-netted the patient to return for follow-up, but again acute deterioration in symptoms precluded onward referral. There were also cases in which investigation results had been received by the practice but were reported with normal results, thereby falsely reassuring the GP as to the patient's condition.

### Patient factors

There were numerous accounts in which patient factors played a significant role in EP. These included patients not presenting to the practice with symptoms related to the EP, although they had consulted during the previous year. In some cases, the patients were known to be infrequent attenders. There were also accounts of patients not re-presenting to the practice after initially consulting with a relevant symptom, or of presenting a considerable amount of time after the initial consultation, in some cases between 15 and 35 weeks. In other cases, the patient refused or did not attend the investigation, or declined earlier referral or admission.

### System issues

There were several instances in which patients had been referred to an outpatient clinic but who ultimately had an EP. This included patients whose 2WW referral had been actioned but who deteriorated before being seen at the clinic, patients who had been seen at the clinic after being referred by 2WW but who were admitted shortly thereafter when their condition deteriorated, and patients who had previously been referred or admitted to hospital but who had been discharged or had been diagnosed with another condition. There were also some examples of patients either waiting a considerable amount of time for an appointment after an urgent referral or of not receiving a clinic appointment at all.

### Opportunities for earlier referral

Although most accounts appear to involve a reasonable explanation for the patient's EP, there were some cases in which an opportunity for earlier referral may have been possible. Examples of this are provided in Box 2.

### Practice learning points related to EP

Not all of the practices identified lessons that they could apply to future cancer diagnoses, a likely reflection of the complexity involved in some cases and the belief that the pathway to diagnosis could not have progressed any differently. However, it was clear from the reflections of others that they had benefited from undertaking the SEA. The learning points described in relation to EP addressed themes common to those identified from previous SEA evaluations (Mitchell *et al*, 2009, 2011).

### Theme 1: presentation and diagnosis

Learning points around presentation and diagnosis considered the inevitability of some EPs, and the atypical nature of others, the need to be vigilant and to consider specific symptoms such as back or shoulder pain in lung cancer (Walter *et al*, 2015), and the use and usefulness of diagnostic tests.

### Theme 2: consultation activity and safety-netting

Unsurprisingly, many of the learning points were based around the consultation and were often framed as safety-netting. They included the importance of ensuring appropriate follow-up, continuity in relation to practitioner seen, good record keeping and being wary of the reassurance provided by diagnostic tests.

### Theme 3: communication and system issues

There was considerable discussion around communication, primarily between primary and secondary care, and also between members of the primary care team. Some reports had highlighted examples of good communication and team working, but there were also instances wherein practices reported that communication could be improved.

### Theme 4: patient factors

Several areas for learning were identified in relation to patient-specific factors, including frequency of attendance and being wary of those who start to use services more regularly, the impact of coexisting morbidity and lifestyle factors, and the need for patient education around symptoms.

### Theme 5: guidelines and referral

The learning points identified around this theme focussed on practices utilising or re-familiarising themselves with guidelines, or on the appropriateness of guidelines and the awareness that many patients did not fit the criteria for referral.

Synthesis of learning points enabled identification of several key recommendations for practice (Box 3).

## Discussion

### Main findings

This study synthesised multiple SEA reports to gain insights into the presentation pathway for patients diagnosed with cancer as a result of an emergency admission. There was extensive primary care input into diagnosis for this patient group, and for the most part any potential ‘delay' in referral could be reasonably explained by the complexity of the presentation or by coexisting patient factors. It would appear that some patients did not experience symptoms before EP or, if they did, did not mention these to their GP when consulting for other things. In other cases, patients presented with symptoms of metastatic disease, and it is unlikely that outcomes for this group would have been altered even if a 2WW referral been made earlier in the process. We did not find any evidence to suggest that patients who present as emergencies are routinely choosing to attend an ED rather than first seeking help from a GP, nor did we find evidence that GPs were routinely referring patients to the ED rather than using the 2WW pathway.

### Strengths and limitations

Although SEA is widely used in primary care practice, applying established methods of analysis to multiple SEAs on a single subject – in this case cancer – to draw inferences about related processes of care is innovative. To our knowledge, such use of SEAs has not been done for other clinical areas. The strength of this work is that it has provided rich, qualitative accounts of what is taking place in general practice prior to EP. In addition, it provides GPs' reflections on the circumstances surrounding each case, along with the lessons learnt from reviewing it, which in turn has enabled the identification of recommendations that are transferrable to other practices. We have previously reported on SEA analysis for lung cancer (Mitchell *et al*, 2009, 2013), and there is some evidence that findings from that work have been used to change practice (Hodges *et al*, 2010). This demonstrates the power of SEA as an educational tool, not only in individual practices but also for shared learning across practices and potentially between primary and secondary care.

Qualitative analysis was applied to data provided by a group of self-selecting practices, and as such we may be presenting the best of primary care practice in England rather than the average. However, this can be said of almost all qualitative work involving practitioners, and, indeed, perhaps obtaining and disseminating learning points from the most reflective practitioners is of considerable advantage. In addition, although practices were asked to provide the most recent relevant case, it was not possible to confirm that this was done, and as such we cannot be certain that there were not instances where a case that showed the practice in the best possible light was reported. Furthermore, our study only included patients registered with general practices, and we cannot comment on the way in which non-registered patients are diagnosed with cancer and whether this might be via the emergency route.

### Comparison with existing literature

The findings from this work complement the recent *Routes to Diagnosis* study (McPhail *et al*, 2013) by providing additional context to the diagnostic pathway and, as such, a more detailed and potentially more accurate reflection of the origin of emergency cancer presentations. Although the total proportion of patients admitted via GP referral and A&E presentation is relatively similar for *Routes to Diagnosis* and this study (89.8% *vs* 78.4%, respectively), the breakdown between the two pathways is reversed, with the majority of patients in the SEAs being admitted by their GP. This may be explained, at least in part, by our considering ED presentations, which were the result of a direct referral by the GP or of the GP advising the patient to attend, to be part of the ‘Emergency: via GP' group.

Although it is possible that emergency cancer presentations may reflect a different cancer biology and patient response to symptoms, as well as failures of interpretation by health professionals, we found evidence of significant primary care activity for a range of cancers. These findings endorse recent work on colorectal cancer by Sheringham *et al*, 2014, who found that for most patients there is considerable activity in primary care in the months before an EP. It is clear then that primary care is a place where interventions may be directed to potentially reduce the incidence of EP, although those interventions must be targeted beyond the GP alone and need to include patients, diagnostics and systems.

### Implications for policy and practice

This analysis has begun to explain the circumstances surrounding documented poorer outcomes for patients who are diagnosed during EPs, and it would appear that such presentations can be classified into two groups: those that were unpreventable and those in which earlier intervention may have had an impact. In the former, patients present either to primary care or to the ED with symptoms of advanced disease, which may well reflect the biological properties of a fast growing aggressive cancer. In addition, some patients have highly unusual presentations with symptoms unrelated to the bodily system in which the cancer is eventually diagnosed. The limitation of the 2WW referral pathway is that it requires a GP to make a prediction of the likely cancer in order to facilitate speedy diagnosis; this is less likely to be accurate when symptoms are unrelated to the primary cancer. In the latter group, wherein the pathway to diagnosis could be improved, SEA initiatives help practices to identify – and ultimately share – ways in which they can improve their ability to recognise and deal with potential cancer symptoms, and thereby try to reduce the number of EPs.

The cases described in the SEAs synthesised for this study once again demonstrate the challenges involved in recognising potential cancer symptoms in primary care, and the additional complexities related to vague, multiple or seemingly self-limiting symptoms. In addition, they highlight the fact that, despite appropriate action being implemented, disease progress can overtake investigation arrangements or indeed the referral process. It should be remembered that an EP is not always a failure of primary care or an inappropriate outcome. For patients whose cancer first presents with or rapidly deteriorates to symptoms such as bowel obstruction, fits, pancytopenia or stroke symptoms, emergency admission is entirely appropriate. However, reducing the number of avoidable EPs will require a package of interventions that includes not only patient education and ongoing reflection of cancer cases among practitioners but also system change, including timely and appropriate access to diagnostic tests – while still attending to the bigger picture of the patient's overall well-being – and to secondary care services.

## Figures and Tables

**Table 1:** Characteristics of the included diagnoses

**Characteristics**	***n*** **(%)**	**Advanced/metastatic**
All patients	222	105 (47.3)
**Gender**		
Male	107 (48.2)	51 (47.7)
Female	107 (48.2)	52 (48.6)
Not reported^a^	8 (3.6)	2 (25.0)
**Age at diagnosis (years)**		
Range	10–92	
x̄/s.d.	65.4/17.2	
Not reported^a^	2 (0.9)	
**Vital status at SEA**		
Alive	127 (57.2)	53 (41.7)
Dead	87 (39.2)	48 (55.2)
Unknown^b^	3 (1.3)	2 (66.7)
Not reported^a^	5 (2.3)	2 (40.0)
**Cancer group**		
Brain	9 (4.1)	—
Breast	2 (0.9)	2 (100)
Carcinoid	1 (0.5)	1 (100)
Central nervous system	1 (0.5)	—
Colorectal	21 (9.4)	9 (42.9)
Gynaecological	25 (11.2)	15 (60.0)
Haematological	22 (9.9)	1 (4.5)
Head and neck	1 (0.5)	1 (100)
Lung	72 (32.4)	40 (55.6)
Melanoma	2 (0.9)	2 (100)
Sarcoma	2 (0.9)	1 (50.0)
Upper GI	44 (19.8)	19 (43.2)
Urological	11 (4.9)	5 (45.5)
Cancer of unknown primary	9 (4.1)	9 (100)

Abbreviations: GI=gastrointestinal; SEA=significant event audit.

a Information on patient age, sex and vital status was specifically requested in the SEA template but was not provided by some responding practitioners.

b Patient moved out of the practice area following diagnosis.

**Table 2:**
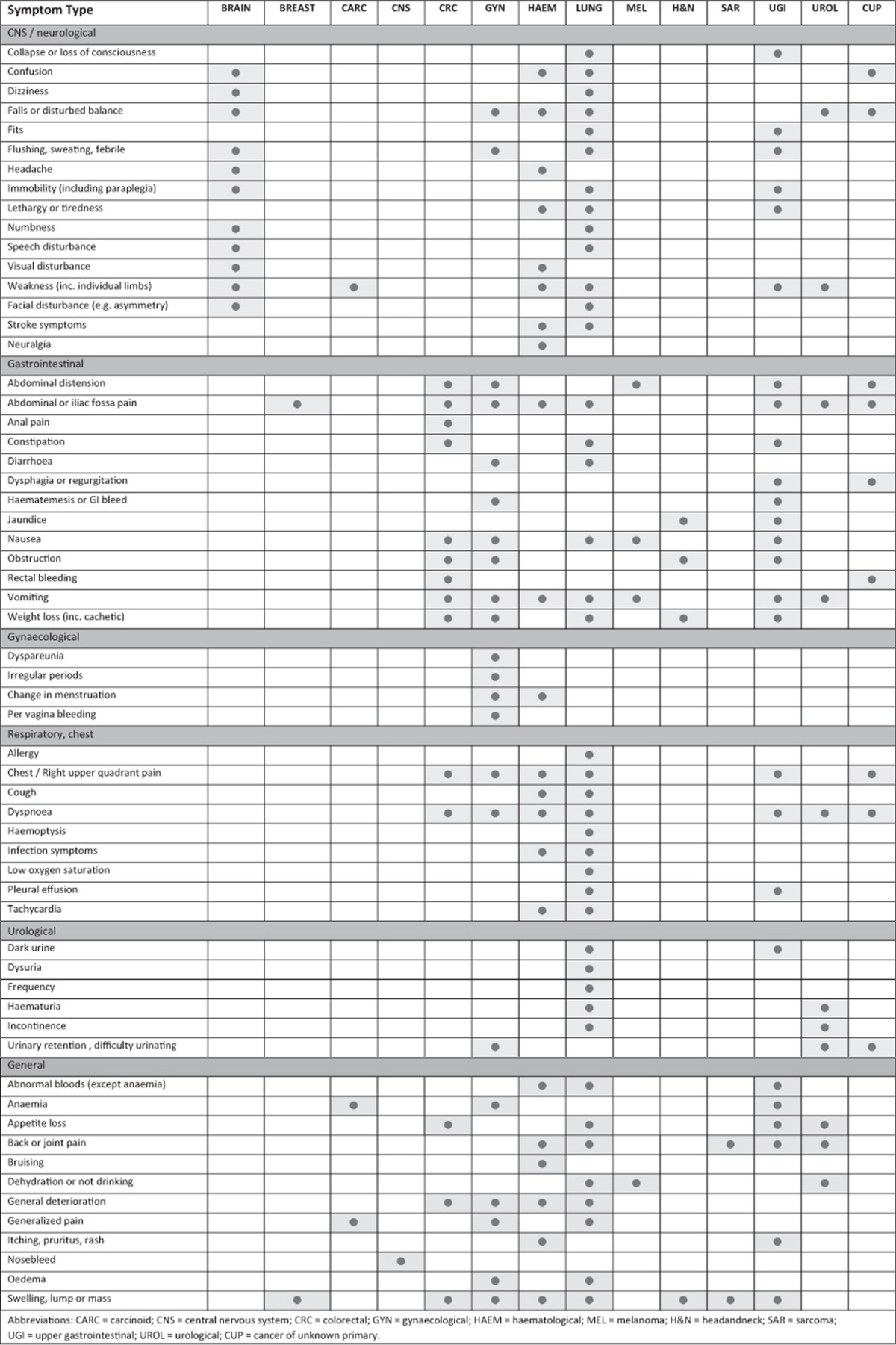
Presenting symptom type by cancer group

**Table 3:** Emergency presentation and primary care involvement

	**Origin of admission (*****n*****, %)**^a^					
**Level of primary care input**	**PC**	**OOH**	**ED**	**OP**	**Other**	**Unclear**
	116 (52.3)	16 (7.2)	58 (26.1)	2 (0.9)	2 (0.9)	28 (12.6)
One-off/short contact^b^	51 (23.0)					
Longer contact	65 (29.3)					
Involved in episode		12 (5.4)	35 (15.8)	2 (0.9)	2 (0.9)	16 (7.2)
Unrelated input		3 (1.3)	19 (8.6)	–	–	7 (3.2)
No previous contact		1 (0.5)	4 (1.8)	–	–	3 (1.3)
Unable to determine		–	–	–	–	2 (0.9)

Abbreviations: ED=patient self-referred or was advised to attend the emergency department; OOH=out of hours; OP=outpatient clinic; PC=primary care.

a Percentages relate to the entire patient population.

b Contact lasting no longer than 2 weeks.

## Box 1. SEA initiatives included in the analysis

- As part of the National Awareness and Early Diagnosis Initiative, a programme of work on cancer diagnosis in primary care was led by the Royal College of General Practitioners. As part of that work, an analysis of SEAs for lung cancer and cancers in TYA was carried out using reports provided by practices from the North of England Cancer Network (2009). A further analysis of UGI and ovarian cancers was carried out using reports provided by practices from the South East London Network (2010). Both analyses were undertaken by EDM with input from UM and GR.
- As interest in differences in diagnosis by care pathway increased, two analyses of SEAs relating to emergency cancer presentation regardless of cancer type were undertaken. The first (in 2012) was based on reports from practices in the North East Yorkshire and Humber Clinical Alliance (Cancer) area. Analysis was carried out by EDM with input from UM. The second (in 2014) used reports from practices in the Yorkshire and the Humber Strategic Clinical Network. Analysis was carried out by EDM with input from UM and LM.
- Identical methods were used in each of the four initiatives, the only difference being the type of patient case that was included – i.e. whether the last patient in the practice was diagnosed with lung, TYA, UGI or ovarian cancer or whether the last patient was diagnosed as the result of an EP.
- This research provides a synthesis of the EP SEAs and includes the following:
  All SEAs from the 2014 initiative-103
  All SEAs from the 2012 initiative-39
  Cases from the 2010 initiative in which the diagnosis was via EP-41 (out of 167)
  Cases from the 2010 initiative in which the diagnosis was via EP-39 (out of 146)
- Although practices were asked to provide one SEA related to their most relevant case, in some instances practices provided more than one. Consequently, the analysis reported here is based on 222 cases from 203 practices. The majority provided a single SEA report (93.4%), while the remainder returned between two and six reports.

## Box 2. Examples of opportunities for earlier referral

- A 66-year-old man presented with symptoms including weight loss 154 days before EP, and was seen a further four times. He was referred to the surgeon for possible diverticulitis and had raised inflammatory markers early in the process, but does not appear to have been investigated further, or had an expedited referral (colorectal).
- A 53-year-old woman was seen four times in 8 weeks with non-resolving chest symptoms. She was treated with antibiotics but does not appear to have been investigated further (lung).
- A 57-year-old man with facial swelling who had 112 days to EP. He was subsequently diagnosed with a metastatic tumour of the right maxillary antrum, and he may have fallen between primary medical care and primary dental care. There were also relevant patient factors (head and neck).
- An 18-year-old man presented with a 12 month history of knee pain; X-ray was arranged and the patient was referred to physiotherapy (X-ray was normal). The patient was seen several times and his inflammatory markers checked, which were also normal. The diagnosis was expedited when the patient found a swelling over the right sacroiliac joint. The patient would appear to have been suffering from referred pain, which, despite the best efforts of the GPs involved to arrive at a diagnosis, did not manifest for some weeks (sarcoma).
- An 82-year-old woman was seen with symptoms suggestive of viral URTI, but blood samples were checked and the patient was found to be anaemic. She was seen the following month with similar symptoms but no action was taken in relation to the low Hb (98 g dl^−1^). She was seen again 1 month later with fatigue; her Hb was checked and was found to be still lower. Before the patient could be seen by a GP, she was admitted with chest pain and diagnosed with stomach cancer (UGI).
- A 63-year-old woman was treated for back pain (thought to be muscular) over a period of 2 months. She then had abdominal pain following NSAID use (abdominal examination at that time was normal) and was prescribed an H2 antagonist. She continued to have back and abdominal pain and was admitted 6 months later with metastatic ovarian cancer (gynaecological).

## Box 3. Recommendations for practice

- Malignancy can be occult despite close follow-up and pursuit of symptoms. As such, emergency admission in some cases is unavoidable and clinically appropriate.
- Consider referral in any patient with unexplained weight loss, new and persistent back pain, or anaemia, even if initial investigations appear normal.
- Be aware of common paraneoplastic phenomenon, which may be the initial presentation of undiagnosed cancer.
- Be wary of infrequent attenders who suddenly have greater contact with the practice.
- Continuity in clinician seen and record keeping is important. Notes should include the ‘plan' and possible diagnosis to aid others who may see the patient.
- Safety-netting is key. Making a follow-up appointment may sometimes be appropriate to ensure that a patient returns if symptoms do not settle.
- Get a ‘second opinion' from colleagues if something looks atypical or advice on appropriate next investigations is needed.
- Be willing to ‘challenge' colleagues' opinions to ensure a holistic approach to patients. Negative investigations should not reassure if the patient does not improve.
- Have practice systems in place to ensure that results, outpatient letters and ED/OOH contacts are seen and dealt with by the appropriate person as soon as possible.
- Carrying out investigations in primary care should not get in the way of a timely 2WW referral.
- Ensure good knowledge of and access to National Institute for Health and Care Excellence guidelines and cancer decision support tools during consultations, but do not allow these to outweigh clinical judgement.
- Take every opportunity to educate the practice population about symptoms that should be discussed with a GP (e.g., notices in waiting areas, and opportunistic enquiries at appointments and screening).
